# A rationale and model for addressing tobacco dependence in substance abuse treatment

**DOI:** 10.1186/1747-597X-1-23

**Published:** 2006-08-14

**Authors:** Kimber P Richter, Julia H Arnsten

**Affiliations:** 1 Department of Preventive Medicine and Public Health, University of Kansas Medical Center, Kansas City, Kansas, USA; 2 Kansas Masonic Cancer Research Institute, University of Kansas Medical Center, Kansas City, Kansas, USA; 3 Division of General Internal Medicine, Department of Medicine, Albert Einstein College of Medicine and Montefiore Medical Center, Bronx, USA

## Abstract

Most persons in drug treatment smoke cigarettes. Until drug treatment facilities systematically treat their patients' tobacco use, millions will flow through the drug treatment system, overcome their primary drug of abuse, but die prematurely from tobacco-related illnesses. This paper reviews the literature on the health benefits of quitting smoking for drug treatment patients, whether smoking causes relapse to other drug or alcohol abuse, the treatment of tobacco dependence, and good and bad times for quitting smoking among drug treatment patients. It also presents a conceptual model and recommendations for treating tobacco in substance abuse treatment, and provides references to internet and paper-copy tools and information for treating tobacco dependence. At present, research on tobacco treatment in drug treatment is in its infancy. Although few drug treatment programs currently offer formal services, many more will likely begin to treat nicotine dependence as external forces and patient demand for these services increases. In the absence of clear guidelines and attention to quality of care, drug treatment programs may adopt smoking cessation services based on cost, convenience, or selection criteria other than efficacy. Because research in this field is relatively new, substance abuse treatment professionals should adhere to the standards of care for the general population, but be prepared to update their practices with emerging interventions that have proven to be effective for patients in drug treatment.

## Review

### Tobacco dependence among drug treatment patients is prevalent and deadly

Globally, millions of people have alcohol and illicit drug use disorders [1,2]. In the U.S. alone, over a million people receive treatment for drug misuse annually [3]. Although no strong population-based data are available, rates of smoking reported in various drug treatment settings range from 93% among outpatient methadone patients in the U.K., to 90% among alcoholic inpatients in the U.S., to 83% among urban methadone maintenance patients in the Northeastern U.S., to 77% among methadone maintenance patients in the Midwestern U.S [4-7].

Until drug treatment facilities systematically treat their patients' tobacco use, millions will flow through the drug treatment system, overcome their primary drug of abuse, but die prematurely from tobacco-related illnesses. For example, among 550 methadone patients in a U.S. Midwest city, the cigarette smoking prevalence rate was 74% even though they had been in treatment an average of 10 years [5]. Current and former smokers from this pool of drug-using patients attributed a number of health and safety problems to their tobacco use, including emphysema and bronchitis, not having enough stamina for work or children, slower recovery from illness, and house fires [8]. Longitudinal research bears this out: a 3-year longitudinal study of 254 poly-drug users in treatment found that rates of disability were significantly higher among smokers than nonsmokers [9].

Although few data are available, it is likely that drug users in recovery experience devastatingly high rates of tobacco-related deaths. In an 11-year retrospective cohort study of 845 persons who had been in addictions treatment, Hurt and colleagues [10] found that 51% of deaths were due to tobacco-related causes. This high rate of tobacco-related mortality is twice that expected in the general population. Likewise, Hser and colleagues [11] found that cigarette smoking contributes to mortality above and beyond deaths due to opiate use; in their 24-year follow-up of drug users that were admitted to drug treatment in 1964, the death rates of smokers were four times that of non-smokers.

### Will drug treatment patients who quit live longer, healthier lives?

One in two long-term smokers will die of a tobacco-related illness [12]. Within the general population, people who stop smoking, even at the age of 40 or 50, avoid more than 90% of the lung cancer risk associated with tobacco [13]. Quitting smoking immediately reduces risks for cardiovascular disease and cancer; it also reduces risks for low birth weight, respiratory illness, and sudden infant death syndrome among the children of smokers [14]. Few data are available on the health effects of quitting smoking specific to drug users in recovery, but drug treatment patients who quit smoking have been shown to improve their quality of life [9]. Regardless of their age, drug treatment patients will probably live longer if they quit. People who stop smoking at the age of 35 gain an estimated 6.1–8.5 years of life; those who quit at the age of 65 live 1.4–3.7 years longer than if they had not quit [15].

### Most drug treatment patients who quit smoking do not relapse to other drug abuse

Drug treatment patients who are able to quit smoking, or who participate in smoking cessation interventions, on average do not relapse but actually have better drug treatment outcomes. Several studies have found that tobacco abstinence correlates to drug abstinence. Lemon and colleagues, [16] in a retrospective study of 2,316 clients enrolled in the U.S. Drug Abuse Treatment Outcomes Study, found that smoking cessation was associated with drug abstinence one year following admission for drug abuse treatment. In another longitudinal study [17] of poly-drug users, drug-positive urine tests among stable smokers were 2–5 times more frequent than drug-positive urine tests among stable non-smokers at all three annual assessment points. In a recent randomized controlled trial of a smoking cessation intervention among methadone patients, patients provided significantly more opiate and cocaine-positive urine tests during times they were smoking cigarettes than when they were tobacco-abstinent [18]. These findings suggest that abstaining from smoking doesn't automatically throw patients into relapse, but they may also be due to self selection – i.e., individual patients who are able to quit smoking are also better able to cope with their other addictions.

A cluster-randomized trial of a smoking cessation intervention among patients with alcoholism offers stronger evidence that treating smoking does not cause relapse. Twelve residential treatment centers in the Midwest were matched and randomly assigned to intervention or control conditions. The intervention condition consisted of one pre-discharge individual counseling session delivered by site staff, and 3 post-discharge counseling sessions delivered by research staff over the phone. Pharmacotherapy for nicotine dependence was not provided to any participants. Participants in the control group received usual care. The intervention was ineffective for smoking cessation: participants in the intervention group were no more likely than participants at control sites to be abstinent from smoking 6 or 12 months after the intervention. More importantly, at both follow-up points, participants at intervention sites were significantly more likely to be abstinent from alcohol than control site participants [19]. Hence, the quit smoking intervention did not undermine, but actually promoted, alcohol abstinence.

However, a subset of people in recovery may be at increased risk for relapse when trying to quit. During the course of another smoking cessation trial among 300 persons recruited from the general community, 9 participants developed such severe psychiatric problems that they were advised to resume smoking [20]. Eight of these participants had a history of depression. There is no way to know how many of these participants would have developed these problems during this time even if they had not been in the trial. Given the prevalence of diagnosed and, more importantly, undiagnosed depression [21], monitoring drug treatment patients for depression and relapse to drug abuse during tobacco quit attempts is warranted.

Taken together, studies on drug relapse and smoking cessation suggest that most drug treatment patients will benefit from trying to quit smoking; most will not relapse to drug abuse as a result; a very small percentage may be at risk for developing either depressive symptoms or drug relapse when trying to quit; and that, during smoking cessation attempts, close monitoring of patients for emergent symptoms of mental illness is crucial.

### Drug treatment patients are willing and able to quit smoking

Recent studies suggest that drug treatment patients are interested in quitting smoking, have tried to quit repeatedly, and often have made a serious attempt to quit within the last year [22-26]. For example, two separate regional surveys in U.S. methadone maintenance clinics found that most patients (70%–80%) were either somewhat or very interested in quitting and that most (68%–75%) had tried to quit at least once in their lives [5,7] One of these studies examined readiness to change and found that nearly half (48%) of respondents were currently contemplating quitting and another 22% were preparing to quit [7]. In a separate study, 40% of clients for whom heroin was the drug of choice indicated they were interested in stopping smoking when they presented for treatment [25].

Epidemiological studies find that people with former and current substance abuse problems *can *quit smoking. People with a past history of alcohol problems achieve the same quit rates as people with no history of alcohol problems [27]. In a national sample of 1,465 active illicit drug users in the U.S., 1 in 5 (21%) were *former *smokers [28]. Two separate regional surveys found that sizable minorities of methadone patients (11%–12%) were former smokers [5,7]. Although the quit rates among drug users are low compared to quit rates in the general population or among alcohol users, there are substantial data showing that substance abuse patients can indeed quit smoking. The question that remains is how best to help even more substances abusers achieve lasting abstinence.

### Substance abuse treatment community response

Some substance abuse treatment facilities are beginning to treat nicotine dependence. In 1998, the SAMHSA Uniform Facility Data Set found 1 in 5 substance abuse treatment facilities offered some form of smoking cessation services [29]. A more recent (2001) survey of Canadian drug abuse treatment facilities found that 54% reported they offered clients help quitting smoking, but only 10% had any formal group or individual therapy dedicated to smoking cessation, and fewer than 1% of facilities offered nicotine replacement or bupropion [30]. A survey of U.S. methadone facilities found that many clinics had provided the following services to at least *one *patient in the past month: 73% provided brief advice to quit, 18% offered quit smoking classes/groups, and 12% prescribed nicotine replacement [31]. However, among methadone clinics that *had *provided service to anyone in the past month, the median number of patients served was small, ranging from 3–20 even though the average clinic size was 229 patients, most of whom smoked. This survey also found that most methadone clinics ban indoor smoking but many allow smoking outdoors, which might send mixed signals to patients regarding the clinic's commitment to treating smoking [32]. A small but significant minority of providers (25%) had discouraged patients from trying to quit smoking [33]. These data suggest that methadone clinics are beginning to address smoking, but that treatment for nicotine dependence is far from routine, few methadone clinics offer standard of care, many have policies that allow outdoor smoking which may undermine treatment, and some actively discourage quit attempts.

### How is tobacco dependence treated?

Effective tobacco treatment is evidence-based and includes office systems that ensure routine intervention and follow-up. Nicotine dependence treatment for the general population has been rigorously evaluated [34-36]. This research has to date focused on delivery of services in medical care settings including outpatient primary care, [37] specialty outpatient clinics, [38] and managed care systems [39]. Based on these studies, the U.S. Public Health Service Guideline recommends that: a) *all *smokers be offered treatment, b) patients *unwilling *to quit be provided with a brief intervention to build motivation, and c) patients *willing *to quit be offered evidence-based treatment [34]. Office-based intervention should follow five major steps (The "*5 A's*") to intervene systematically with patients: *Ask *the patient if she or he uses tobacco; *Advise *him or her to quit; *Assess *willingness to quit; *Assist *with quit attempt; and *Arrange *for follow-up to prevent/address relapse. Model programs in large managed-care organizations suggest that full implementation of the U.S. PHS Guideline increases the use of proven treatments and decreases smoking prevalence [40].

In the U.K., the National Institute for Health and Clinical Excellence (NICE) offers similar guidelines, namely, that all smokers be advised to quit, asked about their interest in quitting, and clearly advised to quit [41]. Perhaps because the U.K. National Health Service offers a universally-available intensive quit-smoking program, it is recommended that health care providers conduct brief interventions with patients, and then offer patients referrals to more intensive support services. Providers who have been trained as National Health Service stop smoking counselors may 'refer' patients to themselves where appropriate.

Pharmacotherapy for smoking cessation consists of 7 first-line medications: bupropion SR, varenicline, and five forms of nicotine replacement therapy (NRT; patch, gum, lozenge, nasal spray, and inhaler). All have been approved for use in treating smoking cessation by the U.S. Food and Drug Administration. Six are recommended for smoking cessation by the National Institute for Clinical Excellence (NICE) in the U.K. The newest, varenicline, is undergoing NICE appraisal for clinical and cost effectiveness. Bupropion and NRT increase the odds of quitting 1.5 to 2 fold over counseling alone [42]. Because bupropion and NRT are equally effective, the choice of which product to use should be guided by patient preferences and the product's adverse effect profile [43]. Excellent descriptions of contraindications, side effects, dosage, duration, and availability of NRT and bupropion are available from several internet sources [44,45].

Bupropion and NRT are more effective than using no medication to quit, but 4/5 of smokers that use any of these medications to quit will return to smoking within a year [46]. A 12-week course of varenicline (2 mg per day) was shown to be more effective for smoking cessation than 12 weeks of bupropion (300 mg per day) in one clinical trial [47]. Two trials that compared daily doses of 300 mg versus 150 mg bupropion for smoking cessation found significantly higher quit rates for 300 mg at end of treatment. Quit rates for the 300 mg dose remained higher but were not significantly different than quit rates for the 150 mg dose at 12 months [48,49]. Varenicline has fewer side effects and contraindications than bupropion, but has not been tested among people in recovery. Bupropion has been shown to be effective for smoking cessation among people with a history of alcoholism [50], and in conjunction with NRT is a promising cessation regimen for methadone patients [51]. Bupropion is also an antidepressant, although its efficacy for smoking cessation is independent of history of depression [50] and is not due to diminished post-cessation depression [52]. However, bupropion may confer some beneficial side effects on depressive symptoms among drug treatment patients, who experience higher rates of depression and depressive symptoms than found in the general population [53]. Patients experiencing adverse side effects at 300 mg can try the lower dose, which is associated with fewer adverse effects and similar quit rates at 12 months. Hence, even though varenicline is newer, potentially more effective for smoking cessation, and has fewer contraindications, bupropion remains an important option for many drug treatment patients because it is less expensive and may confer ancillary mental health benefits.

Behavioral treatment for nicotine dependence typically consists of group or individual counseling and pharmacotherapy. Counseling of greater than 10 minutes produces significantly greater cessation rates when compared to no-contact interventions – at least 4 sessions are recommended [34]. Medications, including bupropion and nicotine replacement therapies, double quit rates when compared to placebo [43,45]. The highest abstinence rates are achieved when pharmacotherapy is combined with intensive counseling [54].

Government guidelines also recommend office-based systems to identify, track, and follow-up with smokers at every visit and remind providers to intervene [34,41]. Without these systems, providers' practices do not change, even when they have participated in training [55,56].

### Do smoking cessation interventions for alcohol and drug treatment patients work?

A number of studies suggest that nicotine dependence treatment can be effective for addictions patients. Studies conducted in alcohol treatment achieved moderate but significant long-term success. Two definitive trials in the U.S. provided patients in treatment for alcohol dependence with nicotine replacement therapy and counseling, and achieved 12-month quit rates of 12% [57,58].

Smoking cessation treatment studies among people in recovery from illicit drug use have achieved impressive quit rates during the intervention, but modest long term outcomes. In two small trials that did not use pharmacotherapy, all patients relapsed at the end of treatment [59,60]. In an uncontrolled trial among 40 patients given 10 weeks of nicotine patches and counseling, 30% of patients at week 4 and 7% at week 12 were tobacco abstinent [61]. The two large-scale trials (N = 383; N = 175) that have been conducted to date compared less intensive to more intensive behavioral therapies, with all participants receiving nicotine patches. These trials, which were both conducted among methadone patients, achieved high rates of both setting a quit date (81%) and abstinence (36%) during treatment. Following treatment, however, most participants quickly relapsed to smoking. At 6–12 months post-intervention, neither trial found significant differences between groups, and across groups abstinence rates were only 5–7% [18,62]. A recent pilot study of an intensive intervention (7 weeks of bupropion, 12 weeks of nicotine gum, and 6 sessions of Motivational Interviewing-based counseling) among 28 methadone patients achieved a quit rate of 14% at 6 months [51].

Patients with co-occurring substance abuse and mental illness warrant heightened surveillance. One study found that smokers with a history of major depression who abstained from smoking were at higher risk of a new major depressive episode than persons with a similar history who continued to smoke [63]. Given the very strong associations between substance abuse and major depression in the general population [64], it is important to screen smoking cessation candidates for history of major depression prior to quit attempts, consider proactively treating persons with a positive history of major depression, and monitor for emergent symptoms during smoking cessation attempts. Substance abuse patients with co-occurring mental illness that wish to quit smoking should be medically evaluated and followed, preferably by a psychiatrist.

### When is it inadvisable to quit smoking?

To date, few studies have addressed whether there are times or situations during which it is inadvisable for drug treatment patients to quit smoking, but the timing of smoking cessation intervention may be important. One trial compared tobacco treatment immediately versus tobacco treatment 6 months after admission to drug treatment [65]. All patients who indicated they were ready to quit smoking received free nicotine replacement therapy. Both groups had equivalent smoking cessation rates, but the group that started smoking cessation treatment on admission had higher rates of alcohol relapse. However, this study did not include a no-treatment control arm to assess alcohol relapse rates among patients receiving no tobacco treatment. Nonetheless, this study suggests that delaying treatment until patients are stable in recovery, perhaps as long as 6 months, may be better for some patients. The one other trial (N = 36) that specifically examined the timing of tobacco treatment in this population found that timing did not significantly impact smoking or alcohol use outcomes [66]. Additional studies examining the impact of concurrent versus delayed nicotine dependence treatment for patients entering substance abuse treatment should be conducted before completely rejecting concurrent treatment [67].

In another study, 408 U.S. methadone treatment providers were surveyed about good and bad times for quitting smoking [33]. Most (38%) clinic leaders thought the best time to treat patients for nicotine dependence was whenever the patient wanted treatment. One in four clinic leaders reported they or one of their staff had ever advised a patient to delay quitting smoking cigarettes. Some reasons for advising delay were: a) alcohol and illicit drug use are the treatment priority, b) patients should not change too many things at one time, c) patients were new to drug treatment, d) patients were experiencing stress, and e) patients were reducing their methadone dose or were "detoxing" to end treatment.

Treating tobacco dependence in drug treatment patients is warranted. Smoking is so deadly, and so many people in drug treatment smoke, that even low rates of cessation will achieve significant improvements in the health of drug users. Tobacco treatment may also improve drug treatment outcomes, regardless of whether patients successfully quit smoking on any one quit attempt. The fact that smoking cessation trials among illicit drug users achieved respectable within-treatment quit rates, but that most patients resumed smoking at the end of treatment, suggests that short term counseling and pharmacotherapy work. However, this approach may not be sufficient for long-term success among drug users. Dual pharmacotherapy and intensive counseling may be more effective, but more research is needed to determine how best to treat nicotine dependence among drug users.

### Implications for delivering high-quality tobacco treatment to drug treatment patients

These studies highlight aspects of treatment that are important for drug treatment patients. It may be best to delay treating patients for tobacco dependence until they have finished induction into drug treatment, perhaps even 6 months of drug treatment [68]. It is important to discuss past experiences with quitting with patients, to assess whether they had emergent symptoms of mental illness or drug relapse during prior quit attempts. During treatment for nicotine dependence patients should be monitored frequently for early symptoms of relapse or mental illness, and clinicians may consider proactively treating such symptoms before a tobacco quit attempt [57]. Drug treatment facilities should avoid undermining quit attempts through smoking policies that send mixed messages, or through staff that discourage patients from trying to quit. Pharmacotherapy is crucial to effective tobacco treatment. Lastly, drug treatment patients may require more intensive, longer-term treatment for nicotine dependence to achieve long term tobacco abstinence. We do not know of studies that provide evidence that smoking cessation is contraindicated for any specific groups of drug treatment patients. This may, unfortunately, be due to a lack of studies on this important topic. Future research should be conducted to understand the impact of smoking, identify better cessation methods, and identify the beneficial and adverse effects of quitting smoking within this population.

## Conclusion

### Conceptual model and recommendations for treating tobacco in substance abuse treatment

**Figure 1:**
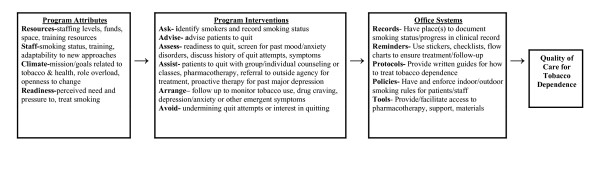
Program attributes, interventions, and office systems for treating tobacco in substance abuse treatment.

A working model (Figure 1) depicts the program attributes and specific interventions that current research suggests will contribute to high-quality tobacco treatment in drug treatment facilities. This model suggests program elements that must be present at the program level to effectively help patients quit smoking.

The program attributes are the four domains of organizational readiness proposed by Simpson et al.'s Texas Christian University Treatment Model [69]. In order to provide high-quality services, facilities must have sufficient resources, staff, climate, and readiness. Resources might include office space, sources for referral, and treatment materials. Staff includes staff time and training. Climate includes favorable attitudes toward treating smoking. Readiness includes motivation to treat smoking, as well as willingness to change clinic infrastructure and practices to provide optimal treatment.

The services that facilities provide should adhere to evidence-based guidelines such as the U.S. PHS Guideline, the U.K. NICE guideline, or other guidelines based on current research. This model employs the *5 A's *for treating tobacco use, modified slightly to incorporate research findings related to smoking cessation in addictions patients. Providers should routinely *ask *about and update patients' smoking status. Patients should routinely be given clear and individualized *advice *to quit smoking. When *assessing *patients' interest in quitting, providers should also document patient's history of mental illness and discuss past quit attempts to assess whether patients relapsed to drug use, felt they were at risk for relapsing, or experienced depressive or other psychological symptoms. Scales such as the CES-D are easily administered and scored and are useful for tracking changes in depressive symptoms [70]. *Assistance *can consist of treatment or referral. The University of Medicine and Dentistry of New Jersey Tobacco Control Program offers a detailed, manualized guide to treating tobacco dependence among drug treatment patients that is based on U.S. PHS guidelines [71]. Pharmacotherapy for nicotine dependence is extremely important for drug treatment patients, as no trials have achieved significant results without it. Providers should *arrange *to follow up with patients on cessation progress, regardless of whether the provider or a referral agency provides treatment. If another provider delivers treatment, arrangements should be made to transfer reports to the addictions treatment provider. Among drug treatment patients, there is a 6^th ^"A" which stands for *Avoid*. Although there may beconditions under which drug treatment patients might be advised against trying to quit smoking, drug treatment programs should adopt policies and practices that do not undermine patients' motivation to quit or sabotage the programs' own smoking cessation interventions.

Office systems that ensure adherence to these treatment guidelines should be developed. Government guidelines stress the importance of office systems for ensuring full and routine implementation of tobacco treatment. Examples of such office supports include written policies and protocols providing rationales and methods for intervention, office forms documenting smoking status and treatment progress, electronic or paper record prompts for intervention and follow-up, routine distribution of self-help materials to smokers, protocols for referring smokers to tobacco treatment specialists outside the facility, procedures for prescribing/obtaining pharmacotherapy for smokers, and periodic record reviews to track facility performance on tobacco treatment among patients [72].

We do not recommend that patients be required to quit smoking, nor that substance abuse treatment be terminated if patients continue to use tobacco. Tobacco use should be treated as a chronic, relapsing condition similar to other forms of drug abuse or other chronic health conditions such as diabetes, asthma, and hypertension [73]. In this approach, the focus is on delivering evidence-based care, monitoring progress, encouraging renewed quit attempts when relapse occurs, and changing tactics if progress is not satisfactory. In accordance with clinical practice guidelines, substance abuse treatment facilities should routinely assess patients' smoking status, routinely assess readiness to quit, routinely offer assistance in quitting smoking, and provide treatment to patients who are ready to quit. Facilities that do not have the resources to treat smoking may refer patients to quitlines or other health care providers that do have specialized training in tobacco treatment.

These are recommendations for what substance abuse treatment facilities should do in order to provide optimal, evidence-based tobacco treatment. Exactly how they achieve or approximate these standards will depend on the resources available to the facility, the smoker, and their community/country. For example, patients in the U.S. and other countries without universal access to health care may have difficulty obtaining quit smoking pharmacotherapy. Many patients in such settings have no health insurance for medications, and their treatment facilities may receive their funding from block grants that must cover the costs of all services. Some of these facilities or patients might be able to access other sources of funding for pharmacotherapy, such as Medicaid (in states in which Medicaid pays for smoking cessation pharmacotherapy). Other facilities might be able to help patients gain access to medications through pharmaceutical drug company medication assistance programs, but some facilities may not be able to provide any access to pharmacotherapy. In other countries, facilities may not be able to provide pharmacotherapy, but patients can readily acquire medications through their health care providers. Many countries, and a few U.S. States, deliver behavioral intervention through referral to tobacco treatment specialists, who work with smokers in person or over the telephone. In these situations, a dual approach might be most appropriate, including a) training/motivating facilities to refer patients and b) developing a cadre of tobacco treatment specialists with expertise in substance abuse treatment.

### More and more facilities will begin treating nicotine dependence

Even in the absence of tailored treatments, or additional resources, broader trends will likely create additional reasons for treating tobacco dependence. Attitudes toward nicotine dependence treatment among the staff of drug abuse treatment programs appear to be changing; surveys conducted in 1999 and 2000 find more staff support for helping patients to quit smoking compared to surveys conducted in the 1980s and early to mid 1990s [74]. In the 1990s, changes in hospital tobacco policy, state laws, and local ordinances required drug abuse treatment facilities to restrict indoor smoking and may have spurred some to begin treating tobacco use [75]. A similar trend is happening in residential and outpatient substance abuse treatment. For example, in New Jersey a state law, staff training, and provision of NRT resulted in widespread adoption of tobacco treatment in residential substance abuse treatment facilities [76]. Disciplinary associations of drug abuse treatment providers such as the American Society for Addiction Medicine (ASAM) and the Association for Drug Abuse Treatment Professionals (NADAAC) now recommend incorporating tobacco treatment into addictions treatment [77,78].

### Adherence to standard of care is essential until optimal care is identified

Research on tobacco treatment in drug treatment is in its infancy – the types of services currently offered have not been well described, few treatment models have been developed for drug users, and measures of services and patient outcomes are lacking. Although it appears that few drug treatment programs offer formal services, many more will likely begin to treat nicotine dependence as external forces and patient demand for these services increases. In the absence of clear guidelines and attention to quality of care, programs may adopt services based on cost, convenience, or selection criteria other than efficacy, similar to treatment choices following the introduction of managed care in drug treatment in the U.S. in the 1990s [79]. For example, 6% of U.S. methadone facilities offer acupuncture for smoking cessation, even though it has not been shown to be effective for helping people quit smoking [75, 80]. Because research in this field is relatively new, substance abuse treatment professionals should adhere to the standards of care for the general population, but be prepared to update their practices with new interventions, proven to be effective for those in drug treatment, as they emerge [34]. The New Jersey initiative suggests that a combination of policy change, free staff training on evidence-based treatment, and governmental provision of pharmacotherapy together can promote widespread adoption of high-quality care [76].

## Competing interests

The author(s) declare that they have no competing interests.

## Authors' contributions

KR conceived of the review and prepared a preliminary draft of the manuscript. Both authors revised drafts of the manuscript and approved the final manuscript.
